# Disparities in healthcare access and utilization among people living with HIV in China: a scoping review and meta-analysis

**DOI:** 10.21203/rs.3.rs-2744464/v1

**Published:** 2023-04-06

**Authors:** Wei Ai, Chengxin Fan, Gifty Marley, Rayner K J Tan, Dan Wu, Jason Ong, Joseph D. Tucker, Gengfeng Fu, Weiming Tang

**Affiliations:** Nanjing Medical University; Nanjing Medical University; University of North Carolina Project-China; University of North Carolina Project-China; University of North Carolina Project-China; Monash University; University of North Carolina Project-China; Jiangsu Provincial Centre for Disease Control and Prevention; Nanjing Medical University

**Keywords:** healthcare disparities, healthcare utilization, quality of healthcare, healthcare needs, HIV, PLWH

## Abstract

**Background:**

Healthcare disparities hinder the goal of ending the HIV pandemic by 2030. This review aimed to understand the status of healthcare disparities among people living with HIV (PLWH) in China and summarize driving factors.

**Methods:**

We searched six databases: PubMed, Web of Science, Cochrane Library, Scopus, China National Knowledge Infrastructure (CNKI), and China Wanfang. English or Chinese articles published between January 2000 and July 2022 were included if they focused on any disparities in access to and utilization of healthcare among PLWH in China. Grey literature, reviews, conferences, and commentaries were excluded. A random effects model was used to calculate the pooled estimates of data on healthcare access/utilization and identified the driving factors of healthcare disparities based on a socio-ecological framework.

**Results:**

A total of 8728 articles were identified in the initial search. Fifty-one articles met the inclusion criteria. Of these studies, 37 studies reported HIV-focused care, and 14 focused on non-HIV-focused care. PLWH aged ≥ 45 years, female, ethnic minority, and infected with HIV through sexual transmission had a higher rate of receiving antiretroviral therapy (ART). Females living with HIV have higher adherence to ART than males. Notably, 20% (95% CI, 9–43%, *I*^2^ = 96%) of PLWH with illness in two weeks did not seek treatment, and 30% (95% CI, 12–74%, *I*^2^ = 90%) refused hospitalization when needed. Barriers to HIV-focused care included the lack of knowledge of HIV/ART and treatment side effects at the individual level, and social discrimination and physician-patient relationships at the community/social level. Structural barriers included out-of-pocket medical costs, and distance and transportation issues. The most frequently reported barriers to non-HIV-focused care were financial constraints and the perceived need for medical services at individual-level factors; and discrimination from healthcare providers, distrust of healthcare services at the community/social level.

**Conclusion:**

This review suggests disparities in ART access, adherence, and utilization of non-HIV-focused care among PLWH. Financial issues and social discrimination were prominent reasons for healthcare disparities in PLWH care. Creating a supportive social environment and expanding insurance policies, like covering more medical services and increasing reimbursement rates could be considered to promote healthcare equity.

## Background

Inequalities undermine global efforts to end the HIV pandemic [1]. New data from The Joint United Nations Program on HIV/AIDS (UNAIDS) revealed that approximately one-quarter of all people living with HIV (PLWH) did not have access to antiretroviral therapy (ART) in 2021 [2]. This proportion is even higher among children living with HIV and those in low- and middle-income countries. For example, in China, more than 26% of children living with HIV aged ≤ 14 years have not received ART as of the end of 2018 [3]. Stigma, discrimination, marginalization of communities, and unequal access to health and other essential services have contributed to increased healthcare disparities among PLWH [4]. In this study, we define healthcare disparities as disparities in access to and utilization of healthcare services among population groups [5–7].

Prompt initiation of ART and retention in HIV care are recognized as key steps in improving clinical health outcomes among PLWH [8]. And the success of the free ART program in China has effectively reduced HIV-related mortality [9]. However, healthcare disparities are still a problem in China [10–12]. For example, a retrospective cohort in Guangxi, China, showed that only 72.1% of people aged ≥ 50 years living with HIV received ART from 1996 to 2019, far lower than younger people (86.1%) [13]. In an era of “treating all”, the coverage of ART among men who have sex with men (MSM) in China was only 60.3% in 2016 [14]. Just 60.1% of PLWH aged ≥ 15 years who injected drugs in China received ART during 2001–2020 [15]. Thus, great disparities exist in access to healthcare among PLWH in China. As life expectancy gets closer to that of the general population, the rising morbidity of non-AIDS-defining diseases (NADs) indicates that PLWH needs more intensive non-HIV healthcare than people without HIV [16]. Details about the overall current situation are not clear, and limited studies have sought to summarize the overall situation in China, although this is important for resolving healthcare disparities in China.

The aims of this study are to 1) understand the status of healthcare disparities among PLWH in China in both HIV-focused and non-HIV- focused care, and 2) summarize the driving forces of healthcare disparities among PLWH in China.

## Methods

### Study design

A scoping review approach was adopted as the research method because it allows researchers to rapidly map out key concepts in a complex research area well based on the main sources of evidence available [17]. The review followed the framework outlined by Arensky & O’Malley (2005) [18] and included five steps: (1) identifying a research question, (2) identifying relevant studies, (3) eligibility screen, (4) charting the data, and (5) collating, summarizing and reporting the results.

### Searching Strategy

We searched and retrieved relevant articles from PubMed, Web of Science, Cochrane Library, Scopus, China National Knowledge Infrastructure (CNKI), and China Wanfang. The search query consisted of terms such as health disparities, health inequities, healthcare disparities, people living with HIV, PLWH, PLHIV, HIV-infected, or other associated terms, which were tailored to the specific requirements of each database. Full search terms, with the respective database, are listed in Additional file 1. Original articles published from January 2000 to July 2022 and published in English or Chinese were eligible for inclusion. All identified articles from the searches were transferred to the EndnoteX9 software tool for managing bibliographies, citations, references, and sharing with other reviewers. Duplicate articles were excluded.

### Eligibility Criteria

All included studies met the following criteria: 1) the study had to be conducted among people living with HIV in China, 2) focused on disparities in health care, including access and utilization of medical services, and 3) used quantitative, qualitative, or mixed method study designs. To keep the inclusion criteria as broad as possible, dissertations were eligible. However, grey literature, reviews, conference articles, and commentaries were excluded.

### Data Screening and Extraction

Two reviewers (WA and CF) first independently assessed the titles and abstracts to identify relevant records for inclusion following the eligibility criteria. When two reviewers disagreed on article selection, a consensus was sought from the third researcher (WT). Full texts of the eligible studies were retrieved and assessed for inclusion following the same screening method. The following data were recorded from the final included studies independently by the same reviewers using Excel data extraction and synthesis sheets. As data were extracted, a data charting form was collectively developed to map studies based on forms of disparity, and the extracted data included, author name (s), year, region, study population, sample sizes, study design, factors associated with healthcare access and utilization (like types of healthcare, variables, and data), and facilitators and barriers of healthcare disparities.

### Data Analysis

Quantitative data on health care access/utilization among PLWH were pooled using a random-effects model in a meta-analysis utilizing R-Language Version 4.1.1. The rate of receiving ART was defined as the number of people receiving ART in his/her survey period divided by the total number of people surveyed. Full ART adherence was defined as participants not missing a dose of antiretroviral drugs in his/her survey period. The 95% and above ART adherence was defined as participants taking ≥ 95% of the dose of antiretroviral drugs in his/her survey period. Higher than 90% ART adherence was defined as participants taking > 90% of the dose of antiretroviral drugs in his/her survey period. The two-week morbidity rate was defined as the number of persons who had a disease in the past two weeks before his/her survey divided by the total number of people surveyed [19]. The two-week visit rate was defined as the number of people who utilized outpatient services in the past two weeks before his/her survey divided by the total number of people surveyed [19]. The non-visit rate of people with illnesses in two weeks was defined as the number of people with illnesses but without medical consultation in the past two weeks before his/her survey divided by the number of people with illnesses in the past two weeks before his/her survey [19]. The annual inpatient rate was defined as the number of people hospitalized in the past year before his/her survey divided by the total number of people surveyed [19]. The rate of non-hospitalization among those who required hospitalization was defined as the number of people who required hospitalization but were not hospitalized divided by the number who required hospitalization [19]. In addition, the driving factors of healthcare disparities were classified into three categories based on a socio-ecological framework: individual level, community and interpersonal level, and structural level [20]. Individual-level factors refer to biological and other personal characteristics, such as knowledge, behavior, income, health history, etc. Community and social level factors are characterized as related to the individual’s social networks and life environment. Structural factors include cultural and social norms and health, economic and social policies.

## Results

### Overall characteristics of the included studies

Figure 1 is the PRISMA flow diagram. The initial search generated a total of 8728 articles. After excluding duplicate references, 7291 articles remained. A review of titles and abstracts revealed that 6669 articles were irrelevant or not conducted in China, leaving 622 articles for full-text screening. Of these, 571 articles did not meet the inclusion criteria because they were review/comments (n = 47), full texts were not found (n = 31), reported wrong outcomes (n = 221), reported the wrong population (n = 28), overlapped data (n = 14), or conducted in other countries (n = 230). The final records consisted of 51 articles.

**Figure 2:**
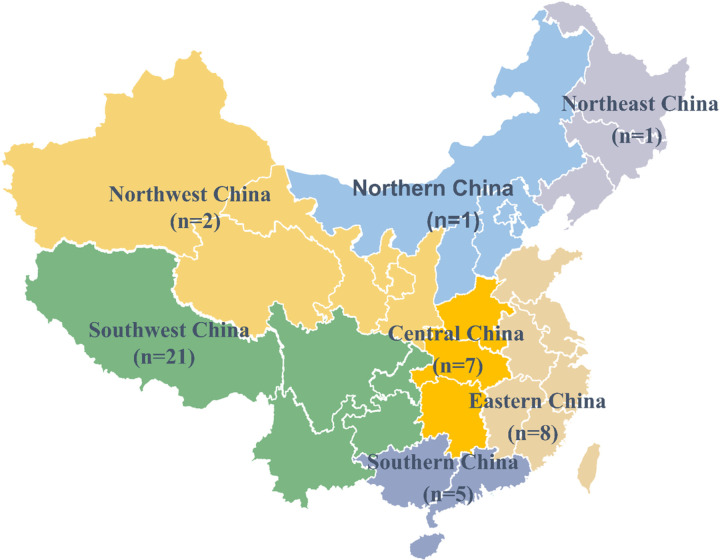
The distribution of study regions

Table 1 describes the characteristics of included articles. The articles reviewed were published from 2000 to 2022, with the majority of articles published after 2010 (n = 41, 80.4%). Figure 2 displays the distribution of study regions. Studies were carried out across seven regions of China: eastern China (n = 8), northern China (n = 1), southern China (n = 5), central China (n = 7), southwest China (n = 21), northwest China (n = 2), northeast China (n = 1). The remaining six studies were conducted in multiple regions. Thirty-seven of the 51 studies were quantitative studies, 7 were qualitative studies and 7 used mixed methods.

### Healthcare access and utilization

**Figure 3:**
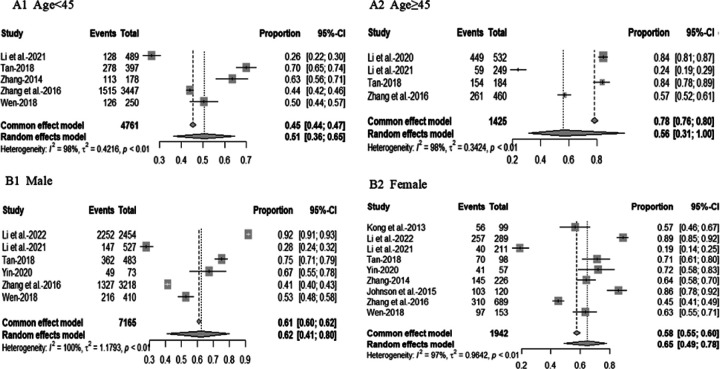
Forest plot of PLWH receiving ART by age and gender. A1 shows a forest plot of receiving ART among people aged <45 years living with HIV. A2 shows a forest plot of receiving ART among people aged≥45 years living with HIV. B1 shows a forest plot of receiving ART among men living with HIV. B2 shows a forest plot of receiving ART among women living with HIV.

**Figure 4:**
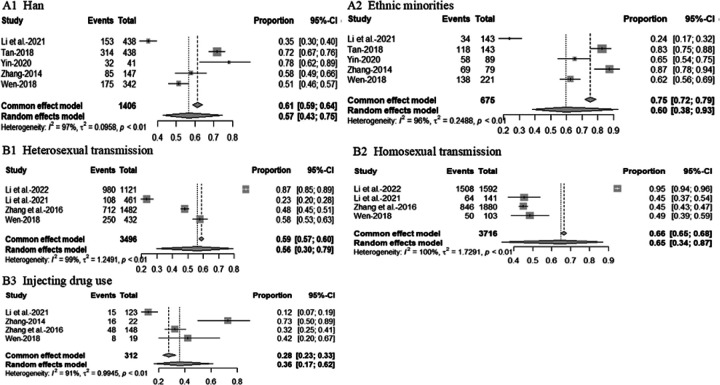
Forest plot of PLWH receiving ART by ethnicity and transmission route. A1 presents a forest plot of receiving ART among PLHIV of Han ethnicity. A2 presents a forest plot of receiving ART among PLHIV of ethnic minorities. B1 shows a forest plot of receiving ART among people infected with HIV through heterosexual transmission. B2 shows a forest plot of receiving ART among people infected with HIV through homosexual transmission. B3 shows a forest plot of receiving ART among people infected with HIV through injecting drug use.

**Figure 5:**
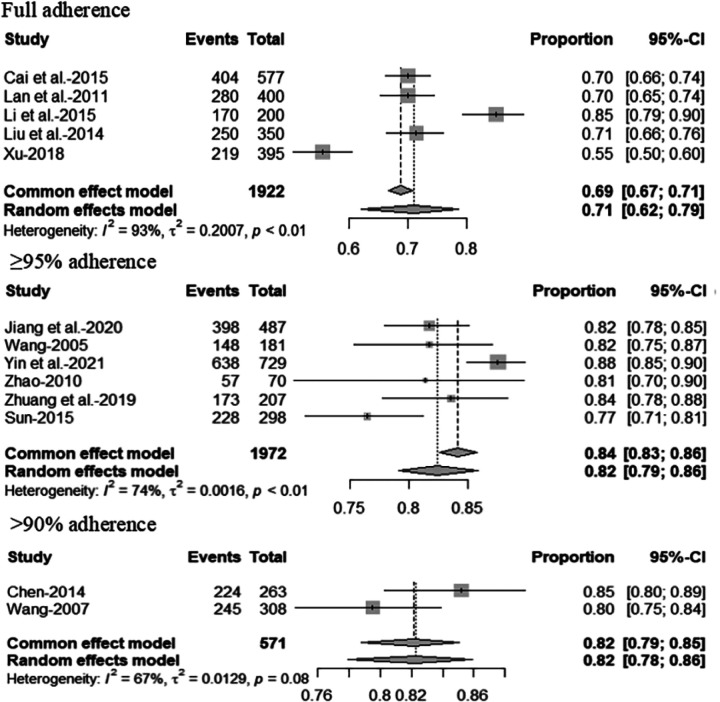
Forest plot of ART adherence among PLWH.

**Figure 6:**
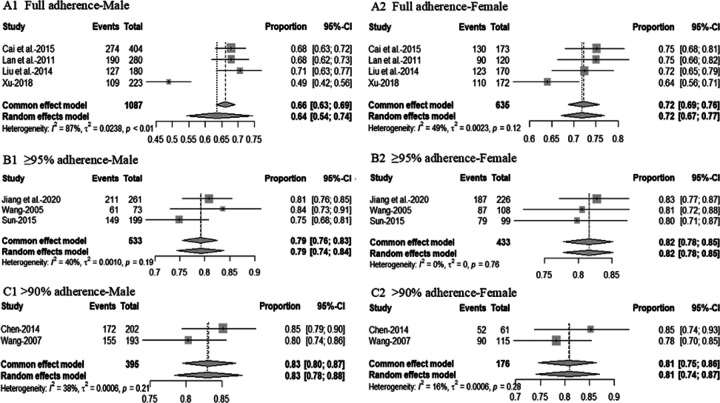
Forest plot of ART adherence among PLWH by gender. A1 shows a forest plot of full ART adherence among men living with HIV. A2 shows a forest plot of full ART adherence among women living with HIV. B1 shows a forest plot of 2:95% ART adherence among men living with HIV. B2 shows a forest plot of ≥95% ART adherence for women living with HIV. C1 shows a forest plot of >90% ART adherence among men living with HIV. C2 shows a forest plot of >90% ART adherence among women living with HIV.

Of the 51 studies that assessed healthcare access and utilization, approximately three-quarters focused on HIV-focused care (n = 37, 72.5%). Of these 37 studies, more than one-third of studies reported on whether participants had received ART (n = 14, 37.8%) (Additional file 2. Table S1). The pooled rate of PLWH aged < 45 years receiving ART was 51% (95% CI, 36–65%, I2 = 98%) and 56% (95% CI, 31%−100%, I2 = 98%) of those aged ≥ 45 years (Fig. 3). The pooled rate of men living with HIV receiving ART was 62% (95%CI, 41–80%, I2 = 100%), and 65% (95%CI, 49–78%, I2 = 97%) of women living with HIV (Fig. 3). The pooled rate of Han ethnic PLWH receiving ART was 57% (95% CI, 43–75%, I2 = 97%) and 60% (95% CI, 38–93%, I2 = 96%) of the ethnic minority (Fig. 4). The pooled rate of receiving ART for PLWH infected with HIV through heterosexual transmission was 56% (95% CI, 30–79%, I2 = 99%), 65% (95% CI, 34–87%, I2 = 100%) of HIV through homosexual transmission and 36% (95% CI, 17–62%, I2 = 91 %) of HIV through injecting drug use (Fig. 4). About half reported ART adherence-related findings (n = 19, 51.4%), with 13 studies reporting adherence using the self-reported method (Additional file 2). Approximately 71% (95%CI, 62–79%, *I*^2^ = 93%) of PLWH reported full adherence, 82% (95%CI, 79–86%, *I*^2^ = 74%) reported ≥ 95% adherence and 82% (95% CI, 78–86%, *I*^2^ = 67%) reported > 90% adherence (Fig. 5). Of those, nearly 64% (95%CI, 54–74%, *I*^2^ = 87%) of men living with HIV and 72% (95%CI, 67–77%, *I*^2^ = 49%) of women living with HIV were fully adherent. About 79% (95%CI, 74–84%, *I*^2^ = 40%) of men living with HIV and 82% (95%CI, 78–85%, *I*^2^ = 0%) of women living with HIV reported ≥ 95% adherence to ART. Approximately 83% (95% CI, 78–88%, *I*^2^ = 38%) of men living with HIV and 81% (95% CI, 74–87%, *I*^2^ = 16%) of women living with HIV reported > 90% ART adherence (Fig. 6). Four studies (10.8%) reported findings of prevention of mother-to-child transmission (PMTCT) services. Table 2shows the pooled rates of receiving ART and adherence among PLWH in China.

Of the 14 studies with data on non-HIV-focused care, 11 studies (78.6%) reported findings of outpatient and/or inpatient healthcare services, 2 studies (14.3%) focused on mental health care, and 1 study (7.1%) on cervical cancer screening (Additional file 3. Table S2). Table 3 shows the pooled rates of non-HIV-focused care among PLWH in China. The two-week morbidity rate of PLWH was 53% (95%CI, 37–68%, *I*^2^ = 98%, n = 7 studies). The two-week visit rate of PLWH was 45% (95%CI, 24–67%, *I*^2^ = 98%, n = 8 studies). The non-visit rate of PLWH with illnesses in two weeks was 20% (95%CI, 9–43%, *I*^2^ = 96%, n = 7 studies). The annual inpatient rate of PLWH was 15% (95%CI, 8–24%, *I*^2^ = 93%, n = 8 studies). The rate of non-hospitalization among PLWH who required hospitalization was 30% (95%CI, 12–74%, *I*^2^ = 90%, n = 4 studies) (Fig. 7).

**Figures 8:**
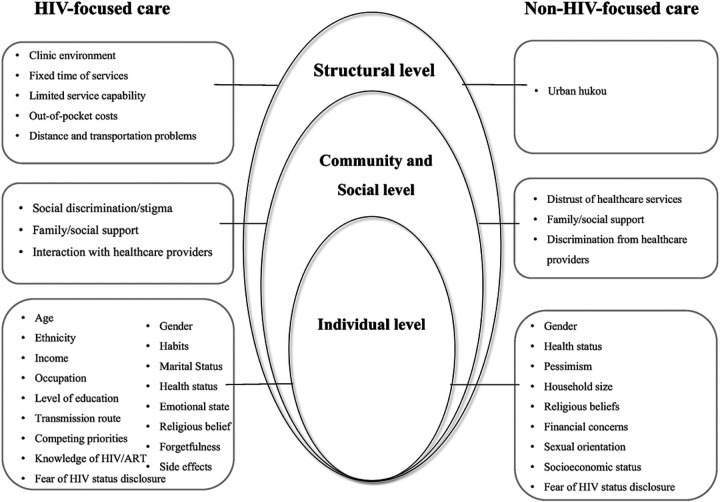
Factors of healthcare access and utilization for PLWH in China.

Fifty-one studies reported factors associated with healthcare access and utilization among PLWH. These factors were often interrelated and exerted their influence at different levels in different contexts. This review identified the factors in each category based on the socio-ecological framework and illustrated them in Fig. 8.

### HIV-focused care

#### Individual level factors

Individual level factors such as age [32, 33, 37, 47], gender [37, 42, 51], ethnicity [26, 27], migration status [23, 24, 27, 53], marital status [24, 33, 36, 50], level of education [23, 33, 36, 50], income [35, 36], employment status [31, 49], personal health [25, 26, 34, 37], knowledge of HIV/ART [21, 25, 27, 29, 31, 34, 35, 40–43, 45, 48, 52, 53, 57], as well as HIV infection factors such as duration of HIV diagnosis [24, 26, 28, 50], were considered to be the driving force of healthcare utilization. The elderly population seemed to have more difficulty accepting and following the antiviral therapy drug regimen [24, 35, 38, 50]. Compared to females, ART adherence was suboptimal among the males living with HIV [46]. Ethnic minority groups were more likely to uptake ART [26]. Evidence on the association between migration and ART adherence was inconsistent. In some studies, migration is a risk factor [23, 27, 53], whereas, in other studies, floating population status is a facilitator[24]. Several studies reported married [24, 37, 50], employed [49], high-education [23, 36], and longer HIV durations [24, 26, 28] groups were more willing to initiate and adhere to ART. PLWH with anxiety and depression [39, 41], smoking [46, 49] and drinking habits [42, 50], religious beliefs [50], and HIV infection through sex [24, 25] or intravenous drug use [21, 32] had a lower likelihood of receiving and adhering to ART. In addition, personal financial constraints [21, 22, 26, 31, 34, 57] and fear of HIV status disclosure [22, 25, 33, 34] caused some PLWH to abandon HIV treatment. PLWH who perceived their health status as good [21, 22, 26, 34, 38] or combined with other diseases [25] or had high CD4 test results [33] tended to refuse or discontinue ART. Going outside [36, 42, 43, 50, 64], forgetfulness [36, 44, 46], side effects of antiretroviral drugs [21, 22, 29, 33–36, 40, 42, 44, 46, 50, 51, 55], lack of time [36, 43], busy work schedules [42, 47, 51], and drug use [42, 44, 46] were also common challenges to daily ART adherence. Alternatively, ART education training [42, 50] and HIV volunteer training [51] can reduce the risk of non-compliance. HIV antibody test too late [23, 30, 55] and lack of awareness of prenatal examinations [54, 56] was the major factor for the failure of prevention of mother-to-child transmission.

#### At the community and social level

At the community and social level, community HIV-related stigma [31] and social discrimination [50–52, 55] were the most frequently cited barriers to HIV care. Social and family support [26, 31, 34, 35, 42, 44, 49, 51] positively affected ART initiation and adherence. Positive interactions with health professionals like confidence in doctors [40, 43, 45, 48], accompanying referrals [25], and reminders from physicians [40, 43, 45, 47, 48] were also a factor for PLWH uptake and adherence to ART. Inadequate recommendations for ART initiation by healthcare providers led to delays in accessing HIV care among some PLWH [29, 33, 34].

#### At the structural level

At the structural level, our findings focused on the health systems and policy. Overall, the clinic environment [51], unfit time of services [53], distance, and transportation problems [38, 42, 53, 57], and limited service capability [57] were the most commonly cited barriers to uptake and adherence. Out-of-pocket medical expenditures [33, 34, 52, 53] also contributed to barriers, and failure to meet the criteria for free ART was the policy barrier for non-ART initiation [21, 25, 61, 64].

### Non-HIV-focused care

The most cited **individual-level barrier** was financial concerns [58–61, 63, 64, 66, 67]. Concern about HIV status disclosure in the course of seeking medical care [59, 62, 71] resulted in medical facility avoidance in some PLWH. Perceived severity of healthcare needs was also associated with healthcare services utilization. People perceiving the symptom or disease not to be serious showed lower odds of seeking healthcare services [58, 61, 64–67]. Male and familial factors, such as a higher number of children and a higher number of people living with HIV in the household, were identified as being associated with less utilization of outpatient and inpatient services [65]. Lack of attention, physical weakness, and pessimism related to HIV infection posed obstacles for female PLWH in cervical cancer screening [71]. PLWH who were homosexual [68] and had low socio-economic status [69] experienced more challenges in utilizing mental health services, while those with religious beliefs [69] were more willing to take mental health assessments.

#### At the community and social level

At the community and social level, PLWH who experienced discrimination from healthcare providers [62, 68] or distrust of healthcare services [59] had a low willingness to seek healthcare services. Support from family members facilitated healthcare utilization[66].

#### Structural factors

Structural factors, such as PLWH from rural areas did not have an urban hukou (household register system), thus they are excluded from urban medical care [68].

## Discussion

Summarizing the status of healthcare disparities among PLWH is essential for improving healthcare equity. This review summarizes literature that examines disparities in healthcare access and utilization among PLWH in China and yielded 51 articles. It extends the existing literature by understanding the status of the disparity, assessing the driving forces of disparities from different levels, and providing evidence for future intervention. Our review found substantial healthcare access and utilization disparities among PLWH in China. We also identified different factors affecting healthcare access and utilization.

In our review, disparities in receiving ART and ART adherence were found among PLWH in China. The rate of receiving ART among PLWH aged ≥ 45 years is higher than those aged < 45 years, which is different from a report from Canada [72]. Avoidant coping, fear of unintentional disclosure, and stigmatizing social norms may diminish the intention of young PLWH to initiate ART [73]. Similar to the South African study [74], we found that the rate of receiving ART is higher among women living with HIV than among men. HIV infection limits the ability to sexually behave, earn money and raise a family, and the resulting stigma undermines treatment seeking for men living with HIV [75]. In addition, people who are infected with HIV through injecting drug use appear to have a lower rate of receiving ART than people infected with HIV through sexual transmission, similar to the finding of a Spanish study [76]. Due to legal challenges, unstable housing, poverty, and lack of social and family support [77, 78], people who inject drugs face multiple barriers to accessing health services, which largely delay ART initiation. The pooled rates of ≥ 95% ART adherence reported by PLWH in China (82%) is higher than that reported in India (77%) as found in a recent systematic review [79]. The various management models such as follow-up management of localization, humanity management, health management and active self-management implemented in China among PLWH have greatly improved ART adherence [80]. Similar to the findings in Zambia and Nepal, adherence to ART was higher among women living with HIV compared to men living with HIV [81, 82]. Masculinity limits the ability of men living with HIV to show vulnerability. Men living with HIV who admit they are sick and seek help can feel their masculinity is compromised, thus they may discontinue treatment [83, 84]. Poverty, stigma, and prioritizing women over men in care also pose barriers to maintaining HIV care among men living with HIV [85].

For non-HIV-focused care utilization, the two-week morbidity rates of PLWH were higher than the national population survey rates in 2018 (32.2%) [86]. HIV weakens the immune system which increases the vulnerability of PLWH to some opportunistic infections and cancers [87]. Moreover, several studies showed that HIV infection also raises the risk of kidney, liver, bone, lung, and cardiovascular diseases [88–90] and may have accounted for our findings. Additionally, the non-visit rate of PLWH with illnesses in two weeks and the rate of non-hospitalization among those who required hospitalization were both higher than the national general population (the non-visit rate of residents with illnesses in two weeks: 1.7%, the rate of non-hospitalization among residents who required hospitalization: 10.2%) [91]. This indicates that access to non-HIV-focused healthcare services in PLWH is inadequate.

Personal financial constraints were the primary cited barrier between the intention to seek care and the actual use of care services. Although the nation-free ART program began in 2003, only PLWH with CD4 cell counts below 200 were eligible for free treatment [92]. The treatment criteria were updated in 2016 enabling all ART for all PLWH to access ART [93]. The strict eligibility criteria in earlier years led some PLWH to pay for ART out of pocket and deterred some from seeking care to date. Also, the costs of laboratory tests and expenditures related to adverse drug events (ADEs) are not covered or subsidized although the free ART policy covers the cost of antiviral drugs for PLWH. This may explain some of the financial constraints cited by PLWH, as a Nigerian study showed that the incidence rate of ADEs among PLWH was high at 28.3% [94]. In addition, the reimbursement rate of medical insurance for the treatment of PLWH is low. For example, medical insurance only covers 12.7% of PLWH outpatient medical expenses in Dongguan City, while in Nanchang City, medical insurance covers 20.8% [95]. Expanded insurance policies, including coverage of more medical services and higher reimbursement rates, are needed to reduce the burden of medical costs on PLWH in China.

Fear of HIV status disclosure was also a common reason for the lack of care engagement and treatment retention. Many people diagnosed with HIV face difficulties in deciding when and how to disclose their status to those around them. The anticipated negative consequences of HIV disclosure are considered an important driver in the disclosure process [96]. Some studies have shown that stigma is a major factor in non-disclosure [97, 98]. Stigma is rooted in culture and driven by personal and social values [99]. HIV-related stigma stems from perceptions of “sexual immorality” and misconceptions about the mechanisms of HIV transmission [100]. In Chinese society, the phenomenon of prejudice against PLWH is widespread. A study among PLWH in central China showed a moderate to high level of perceived stigma [101]. More than 70% of healthcare workers had discriminated against PLWH in the delivery of medical services in healthcare settings [102]. Therefore, interventions to reduce HIV-related stigma such as enhancing social support, improving the quality of HIV care, and strengthening the capacity of healthcare providers to promote the availability of healthcare services among PLWH are necessary.

This review summarizes the status and the driving forces of healthcare access and utilization disparities among PLWH in China and provides directions for future interventions to promote healthcare equity. Creating a supportive social environment and expanding insurance policies could be considered to promote healthcare equity. However, there are some limitations to our scoping review. First, the included articles were published from 2000 to 2022, in which the free ART policy underwent several adjustments. Thus, part of our study before 2016 was based on the old policy, which may reduce the applicability of the results. Furthermore, the pooled rate of adherence estimated in this review may be overestimated as the 13 included studies used PLWH self-reports to estimate adherence. Another shortcoming is that studies included in this scoping review are only related to the Chinese context, which may limit generalizability. Nevertheless, the findings of this scoping review will provide a good basis for further syntheses.

## Conclusion

This review indicated great disparities in ART access, adherence, and utilization of non-HIV-focused care services among PLWH. Financial issues, social discrimination, structural problems in the health care system, and lack of psychological support are prominent in the process of utilizing medical services for PLWH. Expanding current insurance policies, including covering more medical services and increasing reimbursement rates are needed to ease the burden of medical costs for PLWH. Also, the government could negotiate with private insurance to subsidize the cost of covering non-HIV- focused services for PLWH. Creating a supportive social environment is needed to reduce HIV-related stigma and discrimination by using crowdsourcing to develop more innovative and participatory stigma reduction interventions.

## Figures and Tables

**Flgure 1:**
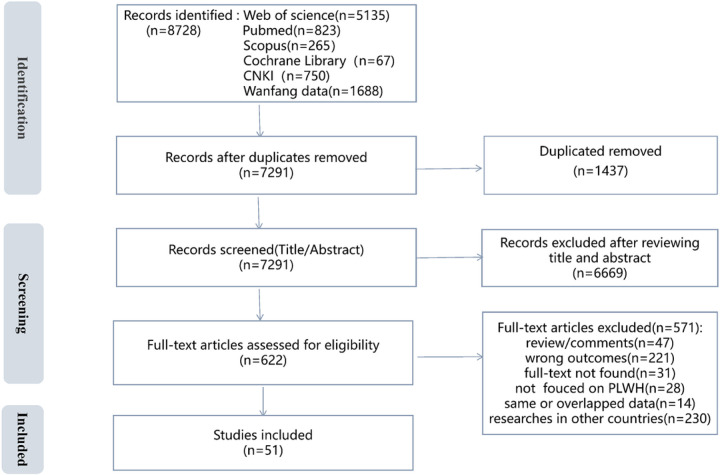
PRISMA flow diagram for included/excluded studies

**Flgure 7:**
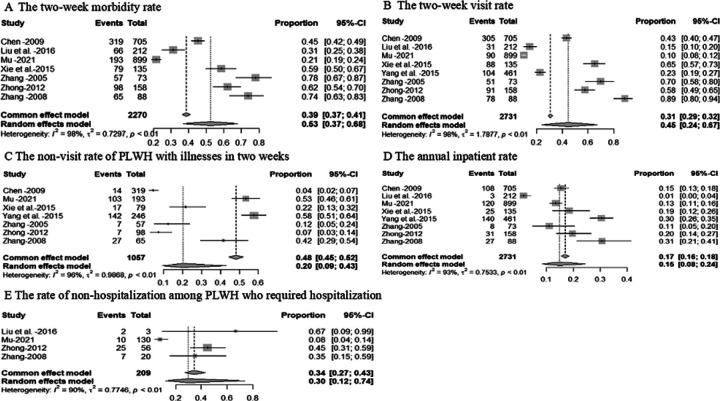
Forest plot of outpatient and inpatient service utilization among PLWH. A presents a forest plot of the two-week morbidity among PLWH. B presents a forest plot of the two-week visit among PLWH. C presents a forest plot of the non-visit rate of PLWH with illnesses in two weeks. D presents a forest plot of the annual inpatient among PLWH. E presents a forest plot of the non-hospitalization among PLWH who required hospitalization.

**Table 1:** General characteristics of included studies, 2000–2022 (n = 51)

Categories	Number (n = 51)	Percentage (%)
**Publication year**		
2001–2005	3	5.9
2006–2010	7	13.7
2011–2015	20	39.2
2016–2022	21	41.2
**Study methods**		
Quantitative	37	72.5
Qualitative	7	13.7
Mixed methods	7	13.7
**Data types** *		
Questionnaire	38	74.5
Medical record review	7	13.7
Interviews	15	29.4
Focus group	2	3.9
**Research timing**		
Prospective	3	5.9
Retrospective	48	94.1
**Study population**		
People living with HIV	35	68.6
People living with HIV in rural areas	1	2.0
Women living with HIV	9	17.6
Children living with HIV	2	3.9
Men who have sex with men	3	5.9
People who use drugs	1	2.0

* where n ≥ 51 as some studies included multiple data types and/or participant groups.

**Table 2:** The pooled rates of receiving ART and adherence among PLWH in China (n = 37)

Categories		Pooled rate
Receiving ART^a^		
Age	<45	51% 95%CI:36–65% *I*^2^ = 98%
	≥45	56% 95%CI:31 −100% *I*^2^ = 98%
Gender	Male	62% 95%CI:41–80% *I*^2^=100%
	Female	65% 95%CI: 49–78% *I*^2^ = 97%
Ethnicity	Han	57% 95%CI:43–75% *I*^2^ = 97%
	Ethnic minorities	60% 95%CI:38–93% *I*^2^ = 96%
Routes of transmission	Heterosexual transmission	56% 95%CI:30%−79% *I*^2^ = 99%
	Homosexual transmission	65% 95%CI: 34–87% *I*^2^ = 100%
	Injecting drug use	36% 5%CI:17–62% *I*^2^ = 91 %
ART adherence		
Full adherence		71% 95%CI:62–79% *I*^2^ = 93%
Gender	Male	64% 95%CI:54–74% *I*^2^ = 87%
	Female	72% 95%CI: 67–77% *I*^2^ = 49%
≥ 95% adherence		82% 95%CI: 79%−86% *I*^2^ = 74%
Gender	Male	79% 95%CI:74–84% *I*^2^ = 40%
	Female	82% 95%CI: 78–85% *I*^2^ = 0%
> 90% adherence		82% 95%CI: 78–86% *I*^2^ = 67%
Gender	Male	83% 95%CI:78–88% *I*^2^ = 38%
	Female	81% 95%CI: 74–87% *I*^2^ = 16%

a ART: Antiretroviral therapy

**Table 3:** The pooled rates of non-HIV-focused care among PLWH in China (n = 11)

Categories	Pooled rate
The two-week morbidity rate	53% 95%CI:37–68% *I*^2^ = 98%
The two-week visit rate	20% 95%CI:24–67% *I*^2^ = 98%
The non-visit rate of PLWH with illnesses in two weeks	20% 95%CI:9–43% *I*^2^ = 96%
The annual inpatient rate	15% 95%CI:8–24% *I*^2^ = 93%
The rate of non-hospitalization among PLWH who required hospitalization	30% 95%CI:12–74% *I*^2^ = 90%

## Data Availability

All supporting data (and its additional files) is attached to this manuscript.

## Abbreviations

HIV: Human Immunodeficiency Virus
UNAIDS: The Joint United Nations Program on HIV/AIDS
PLWH: People Living With HIV
ART: Antiretroviral Therapy
CNKI: China National Knowledge Infrastructure
MSM: Men Who Have Sex with Men
NADs: Non-AIDS-Defining Diseases
PMTCT: Prevention Of Mother-To-Child Transmission
ADEs: Adverse Drug Events
